# Size-dependent chemosensitization of doxorubicin-loaded polymeric nanoparticles for malignant glioma chemotherapy

**DOI:** 10.1080/21655979.2021.2006568

**Published:** 2021-12-11

**Authors:** Meng Gao, Yue Chen, Chenghu Wu

**Affiliations:** a Department of Gastroenterology, The Second People’s Hospital of Hefei, Hefei Hospital Affiliated to Anhui Medical University, Hefei, 230011, China; b School of Clinical Medicine, Anhui Medical University, Hefei, China; c Joint Centre of Translational Medicine, The First Affiliated Hospital of Wenzhou Medical University, Wenzhou, Zhejiang, China

**Keywords:** Malignant glioma, doxorubicin-loaded nanoparticles, chemosensitization, tumor chemotherapy, size-dependent

## Abstract

Chemotherapy is a traditional treatment method in clinical cancer treatment. However, it is limited due to the large toxic side effects of chemotherapeutics. Nanomedicines have shown great potential in the application of tumor therapy. The size of nanoparticles as a crucial factor in the enhanced permeability and retention (EPR) effect can be regulated for the enhanced chemotherapy. Therefore, we believe that regulation of nanoparticle size can be used as an effective sensitizer to enhance the therapeutic effect of chemotherapy drugs on tumors. Here, we prepared several nanoparticles of different hydrodynamic diameters commonly found in nanomedical applications by the diblock copolymer of methoxy polyethylene glycol-poly (ϵ‐caprolactone) (mPEG-PCL). The blood circulation effect and organ distribution in blood were detected by fluorescence labeled nanoparticles. We found that the small-sized nanoparticles exhibited much longer blood circulation time than the large-sized nanoparticles *in vivo*, and thus the nanoparticle concentration in the tumor tissue was relatively high. Systematic injection of the doxorubicin (DOX) loaded nanoparticles can effectively inhibit tumor growth. Compared to the free drug, tumor cells were much more sensitive to DOX loaded nanoparticles of small size. Our nano-drug delivery system has been proven to be safe and nontoxic *in vivo* and was suitable for use as a sensitizer in clinical oncology chemotherapy.

## 1. Introducti on

Malignant glioma has been a relatively common malignant brain tumor, and they rarely metastasize among the aggressive tumors. Patients usually suffer from the disease with a poor five-year survival rate [1]. Currently, chemotherapy has been the most commonly used treatment in the most malignant tumor [2–4]. However, it still hardly exhibits a satisfactory treatment effect of glioma. It also faces the problems of severe side effects of chemotherapeutics, which is also common in other cancer treatments [5,6]. New chemotherapeutic agents seem to be the most effective strategy for the reduction of side effects. It is worrisome the development of a new drug should be tortuous and complex, and no guarantee of the elimination of significant side effects. Considering that the essential cause of side effects is the poor blood circulation and low accumulation in tumor of chemotherapeutic drugs [7–9]. The ideal drug delivery system could improve the sensitivity of tumor cells to chemotherapy drugs, and hopefully enhance the efficacy of tumor chemotherapy [10].

In the past decade, multifunctional nanoparticles can be applied to many aspects of life and affect many things as a powerful carrier system [11–15]. Nanoparticles have shown great promise in drug delivery to treat disease [16–18]. Especially for tumors, nanoscale size facilitates the accumulation of anticancer drugs into tumors by EPR effect [19,20]. At present, there are many materials used in the design and application of nanomedicine, such as metals [21], carbon [22], and polymer [23], demonstrating good therapeutic effect. Therefore, it is greatly believed that nanoparticle-mediated chemotherapy can improve efficacy while reducing side effects [24–26]. However, nanoparticles themselves have many properties that limit the efficiency of drug delivery *in vivo*. For example, size, zeta potential, and surface modification are all inherent characteristics [27–31]. Nanoparticles with different characteristics can significantly affect blood circulation and tumor accumulation in vivo. Insufficient tumor accumulation will essentially weaken the therapeutic effect of chemotherapy drugs, and may lead to drug tolerance of tumor cells. Moreover, low drug concentration in tumor mean that much more chemotherapy drugs distribute into healthy organs, which will inevitably cause significant side effects. Surface modification with polyethylene glycol (PEG) is a commonly used strategy to prolong the nanoparticles circulation time in the blood. Many studies have also confirmed that the nano properties of PEG on the nanoparticles can significantly affect their behavior and fate *in vivo* [32,33]. To deal with these barriers, researchers have developed a variety of nano-systems in terms of tumor and biological characteristics, including morphology control [34], acid responsiveness [35], and enzyme responsiveness [36], to increase drug accumulation in tumor sites and reduce side effects. Although many nano systems have achieved good results in the experimental stage of tumor therapy, the complex preparation process, uncertain reproducibility and harsh experimental conditions limit their application in cancer therapy. Considering the EPR effect of nanoparticle accumulation in tumor tissue, size effect is one of the most important nano characteristic, which can significantly alter EPR effect and the ultimate accumulation efficiency of nanoparticles in tumor site. Therefore, if the regulation of nanoparticle size can realize the long circulation effect in the blood and the high accumulation in tumor sites, it will help to improve the sensitivity of tumor cells to chemotherapy drugs and the efficacy of tumor chemotherapy.

Herein, we aim to explore the effect of nanoparticles of sizes on tumor chemotherapy sensitization. Polymer nanoparticles of different sizes were designed to deliver DOX by the mixture of mPEG-PCL and PCL. Compared to the other widely used materials, PEG and PCL are all the approved biomedical materials of food and drug administration with the potential for clinical translation [37,38]. Nanoparticles of different sizes are prepared by changing the proportions and molecular weights of the polymers with the method of dialysis. In addition, DOX itself can emit fluorescence, which could indicate the biological fate of nanoparticles in the body [39]. Nanoparticles of small size (62 nm) can exhibit the long circulation time and get rid of the lingering effect of main metabolic organ to the greatest extent. Subsequently, they were enriched in tumors by the EPR effect. Therefore, we hypothesize that nanoparticles of small size are effective chemotherapeutic sensitizers. To verify the hypothesis, the tumor therapy with the DOX loaded nanoparticles of various size were conducted. As we suspected, tumors in the small-size nanoparticle treatment group were highly sensitive to the chemotherapeutic drugs of DOX and tumor volume was effectively suppressed. To further guarantee the safe of nanoparticles *in vivo*, the main organs were removed for histological examination. The results of systematic investigation showed that our treatment system did not produce toxic effects on normal organs and can avoid the damage of chemotherapeutic drugs to the organism to the greatest extent. Therefore, our goal is to use small size nanoparticles as chemotherapeutic sensitizers to improve the therapeutic efficacy of cancer patients.

## 2. Experimental details

### 2.1. Materials

The different diblock copolymer of mPEG-PCL were purchased from ZZBIO Co., Ltd (Shanghai, China). Dialysis bags (MWCO = 4000 Da) were purchased from Macklin Biochemical Co., Ltd (Shanghai, China). Doxorubicin was purchased from Solarbio Science & Technology Co., Ltd (Beijing, China). Dulbecco’s modified eagle medium (DMEM) and fetal bovine serum (FBS) were all purchased from ExCell Bio (Shanghai, China). Chloroform, acetonitrile and 4% formaldehyde were purchased from Sigma Aldrich, St. (Louis, USA). DAPI was purchased from Beyotime (Haimen, China). All the reagents were used according to the instructions.

### 2.2. Establishment of malignant glioma xenograft tumor model

U87MG cells were cultured in the DMEM containing 10% FBS and 1% penicillin/streptomycin in carbon dioxide incubator. After three passages, the cells were collected by the 2.5% trypsin. Then, the cell suspensions were centrifugated at 1000 rpm for 5 min to remove the dead cells. For the construction of malignant glioma xenograft tumor model, 2 × 10^6^ U87MG cells were resuspended in the 150 μL 1 × PBS with 10% matrigel. The NOD-SCID mice were injected subcutaneously of the cell suspension. All animals were treated in compliance with the guidelines outlined in the Guide for the Care and Use of Laboratory Animals. The procedures were approved by the Animal Care and Use Committee of Anhui Medical University.

### 2.3. Preparation and characterization of polymeric nanoparticles

Nanoparticles of different sizes were prepared by the dialysis method [30,31]. Firstly, the diblock copolymer of mPEG-PCL and homopolymer of PCL were dissolved in the acetonitrile, then they were mixed at a certain proportion with agitation for 15 min. To promote hydrophilic and hydrophobic self-assembly, four volumes of Mili-Q ultrapure water were added into the mixture quickly with increased stirring speed. The prepared nanoparticles were collected in the dialysis bag placed into the ultrapure water to remove the acetonitrile. Sizes of nanoparticles could be changed by the alteration of the proportion between mPEG-PCL and PCL. Detailed information was shown in Table 1. Dynamic light scattering (DLS) of Malvern Zetasizer Nano ZS90 was used for the characterization of nanoparticle sizes. For the preparation of DOX loaded nanoparticles, an appropriate amount of hydrophobic DOX was addded during the preparation. The detection of fluorescence spectrophotometers showed that the drug loading content of the nanoparticles was between 4% and 7%.

**Table 1.** Composition and characterization of nanoparticles

		Composition				
Nanoparticle	Formulation	PEG-PCL (mg)	PCL (mg)	CL/EG (molar ratio)	Rh (nm)	Zeta potential (mV)
NP_I_	NP_PEG3.4k-PCL4.5k/PCL3.5K_	4	0.3	0.58	38	−4.5
NP_II_	NP_PEG3.4k-PCL4.5k/PCL3.5K_	4	0.65	0.66	58	−5.8
NP_III_	NP_PEG3.4k-PCL5.7k/PCL3.5K_	4	7	2.45	78	−4.1
NP_IV_	NP_PEG3.4k-PCL5.5k/PCL3.5K_	4	3	1.38	104	−3.6
NP_v_	NP_PEG3.4k-PCL6k/PCL3.5K_	4	3	1.48	135.2	−3.9

### 2.4. The pharmacokinetic of nanoparticles in vivo

To accurately understand the circulation differences of various nanoparticles in the blood, enough 8-week female NOD-SCID mice were divided into five groups. DOX-loaded nanoparticles of different sizes were injected intravenously at the dose of 100 μg DOX. Blood was obtained from the mouse's eyes without sacrifice at a pre-determined time. Then, the blood was centrifuged to isolate the serum at 5000 rpm for 5 min. The isolated serum was lyophilized and dissolved in the 100 μL acetonitrile. Many impurities, such as undissolved proteins, were removed by centrifugation. The supernatant was collected for the fluorescence detection by the BioTek Wavelength Microplate (Epoch, America). The pharmacokinetic parameters of half-life, bioavailability and clearance efficiency were calculated by the DAS 3.0 program [31].

### 2.5. The biodistribution of nanoparticles in vivo

The mice bearing U87MG tumors were divided into five groups. The DOX-loaded nanoparticles of various sizes were injected intravenously into the mice. After a certain period of time (6, 12, 24 and 48 h), the mice were sacrificed for organs (liver, kidney, spleen, and tumor mainly) retrieval. The organs were washed with PBS and weighed. The tissues were ground into a homogenate and mixed with 6 mL acetonitrile via oscillators. The mixtures were centrifuged (10,000 rpm, 30 min) at room temperature to collect the acetonitrile containing dissolved DOX. Then, DOX was obtained by removing acetonitrile solvent with rotary evaporator. 200 μL acetonitrile was added into the tube to dissolve the DOX for the detection of BioTek Wavelength Microplate [30].

For the microscopic observation of nanoparticle accumulation in organs, the spleen, kidney, liver, and tumor of the smallest size nanoparticle group were removed at the time point of 72 h for the preparation of tissue sections. Firstly, the organs were washed and fixed in 4% paraformaldehyde overnight. The tissues were cut into 7 μm slices by frozen slicer (Lecia CM3050S, Germany). After being washed three times, the tissue slices were stained with DAPI and Alexafluor 488 phalloidin at room temperature according to the instructions. Then, the accumulation of nanoparticles in organs was visualized by the laser confocal scanning microscope (LCSM, LSM 710, Carl ZeissInc, Germany) [30,31].

### 2.6. Inhibition of malignant glioma growth in vivo

The mice bearing the U87MG xenograft tumors were randomly divided into seven groups when the tumor volume reached 50 mm^3^, namely, PBS, DOX, NP_I@DOX_, NP_II@DOX_, NP_III@DOX_, NP_IV@DOX_ and NP_V@DOX_ group. The mice were injected intravenously with different size nanoparticles at the equivalent dose of 100 μg DOX per mouse. The mice received injection every 2 days for six injections in total. During the treatment, the tumors in mice were monitored by the calipers. The tumor volume can be calculated with the equation of tumor volume (mm^3^) = 0.5 × length × width^2^. Mouse body weight was also recorded during the treatment. At the end of treatment, the mice were euthanized and the tumors were removed for weighing and images.

For the safety evaluation of nanoparticles in vivo, the main organs of heart, liver, kidney, spleen, and lung were taken out for histochemical analysis. The nucleus was stained in blue and the cytoplasm was in red.

### 2.7. Statistical analysis

Data were presented as mean ± s.d. The statistical significance was judged by the Student’s *t*-test; The *P* < 0.05 was considered statistically significant in all analyses (95% confidence level).

## 3. Results and discussion

Size effect is one of the most important nano characteristics, which can significantly alter EPR effect and the ultimate accumulation efficiency of nanoparticles in tumor site. We aim to explore the effect of nanoparticles of sizes on tumor chemotherapy sensitization. We hypothesize that nanoparticles of small size are effective chemotherapeutic sensitizers. Nanoparticles of different sizes are prepared by changing the proportions and molecular weights of the polymers with the method of dialysis. We discuss the changes of their biological behavior *in vivo*.

### 3.1. Synthesis and regulation of the size of nanoparticles

The size of nanoparticles plays an important role in the EPR effect of drug delivery. Suitable size will facilitate the efficient delivery of the drug to the tumor tissue and promote the sensitivity of tumor cells to chemotherapy drugs. Therefore, we developed a simple size-controlling method for the preparation of nanoparticles of different sizes by dialysis method. We adjusted the molar ratio of CL and EG in the preparation system to achieve the different size. As the results shown in Table 1, the sizes of nanoparticles were varied from 37 to 135.2 nm. In addition, the zeta potential of them were all close to the neutral. So far, we have developed a simple method that can easily regulate the size by changing the ratio of hydrophilic and hydrophobic groups without affecting other nano properties, such as zeta potential.

### 3.2. Nanoparticles with the smaller size exhibited long blood circulation

**Figure 1.**
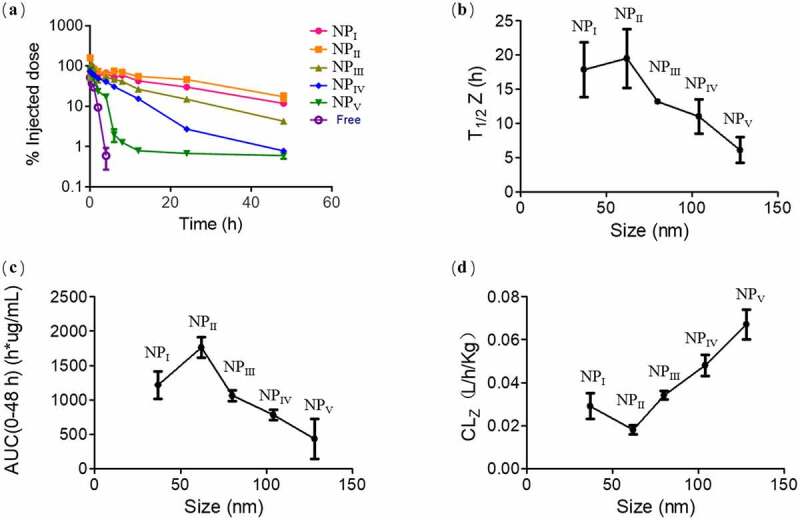
Effect of nanoparticle sizes on the pharmacokinetics. (a) Concentration of nanoparticles with different size in the mice serum after the injection within 48 hours. (b)The terminal half-time (T_1/2_), (c) area under the serum concentration–time curve (AUC_(0–48 h)_) and (d) clearance (CL_Z_) as pharmacokinetics parameters were analyzed. Data were mean ± s.d. from n = 3 independent experiments

The first problem that nanoparticles faced was blood circulation when injected into the body. The nanoparticles with long circulation time will have more opportunities to enrich in tumor tissues through EPR effect. In order to investigate the differences in the circulation of nanoparticles of different sizes in the blood. Mice were injected with sufficient amounts of DOX-loaded nanoparticles of different sizes. At a preplanned time point, the blood of mice was taken out and quantified by fluorescence. It can be obviously seen from the pharmacokinetic curve that more than 50% of the largest nanoparticles have been cleared within half an hour. There were no obvious differences in the short time among other sizes of nanoparticles; however, they began to show obvious differences after 4 hours. The nanoparticles of 104 nm remained almost 1% in blood that similar to the largest nanoparticle of 128 nm over 48 h. Nanoparticles of Other size remain much greater in the bloodstream. The overall phenomenon demonstrated that the larger size of nanoparticles were more likely to be cleared from the blood. An interesting phenomenon was that the nanoparticle circulation time of 62 nm was slightly better than that of 37 nm (Figure 1(a)). It involved many factors such as whether nanoparticles of larger sizes will easily form aggregates to accelerate the clearance in the blood, which required further research in the future. To further demonstrate the differences among them, pharmacokinetic software were used to calculate their various pharmacokinetic parameters according to the non-compartment model. As the results shown in Figure 1(b), the terminal half-life (T_1/2_ response to the circulation time) of 62 nm reach 19.46 h within 48 h which was much higher than other nanoparticles. It was 1.09-fold and 3.18-fold higher than the nanoparticles of 37 nm and 135.2 nm. Another important parameter reflect the circulation effect was the concentration-time cuive (AUC_(0–48h)_). Similar to the results of T_1/2_, the nanoparticles of 62 nm demonstrated the optimal bioavailability of 1760.4 h*μg/mL (Figure 1(c)). The clearance rate (CL_Z_) was another illustration of the circulation differences among the nanoparticles. For the nanoparticles above 62 nm, the clearance rate in blood increased with the hydrodynamic diameter. Nanoparticles of 37 nm were more easily cleared than that of 62 nm. It also explained why the performance of circulation of nanoparticles of 62 nm were better than others (Figure 1(d)). Although such results can be as a guide for the design of nanoparticles in the application, the internal mechanism of action remains to be further explored.

### 3.3. Small size facilitated the accumulation of nanoparticles in tumor tissues

**Figure 2.**
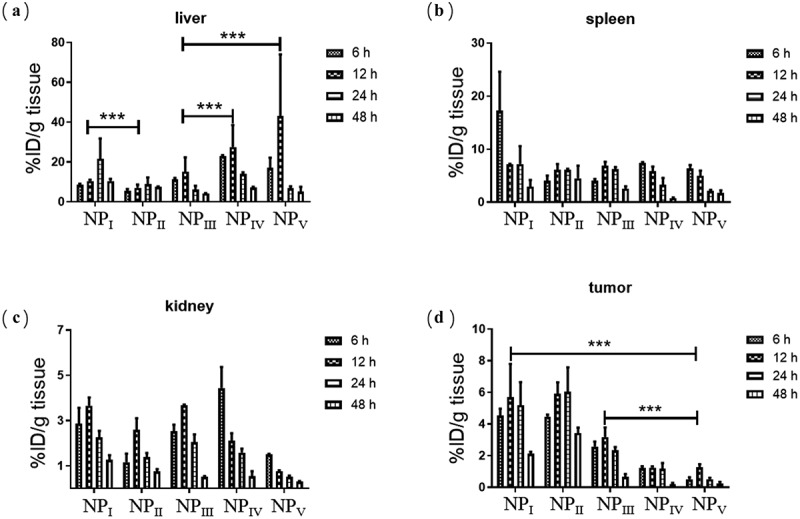
Distribution of nanoparticles with different sizes in liver (a), spleen (b) and kidney (c). Accumulation of various nanoparticles in tumors. (n = 3, data were means ± s.d.) *p < 0.05, **p < 0.01, ***p < 0.001

Nanoparticles with small size could exhibit good circulation effect was confirmed in previous experiments. According to the results, we hypothesized that nanoparticles of small size could avoid the clearance of the main metabolic organs and accumulate effectively in tumor tissues. We evaluated the distribution of nanoparticles with various sizes in the main organs of U87MG tumors beared mice. The liver, spleen, and kidney were the most important scavenging organs of the organism. As shown in Figure 2(a), the content of nanoparticles with the diameter of 135.2 nm in the liver obviously much more than the other nanoparticles. The overall accumulation of different nanoparticles was high in 12 hours. With the proceed of time, the nanoparticles in the liver were metabolized with only a small surplus. However, there were no significant difference among the various nanoparticles in the spleen and kidney (Figure 2(b) and 2(C)). Generally, nanoparticles smaller than 10 nm were mainly cleared by the kidney, which has been relatively clear in the previous research. We believe these can explain why the accumulation of all the nanoparticles in the kidney were so low. As for the reasons of the low retention of all nanoparticles in the spleen, further study was needed. Finally, we isolated tumor tissues from the mice and quantified the nanoparticles. As predicted, the accumulation of small nanoparticles in tumors was significantly higher than that of other sizes, especially nanoparticles of 62 nm. Even after 48 hours, more than 3.6% of the nanoparticles remained in the tumor tissue (Figure 2(d)). The above experimental results partly explain the differences in the enrichment of various nanoparticles from the perspective of clearance.

**Figure 3.**
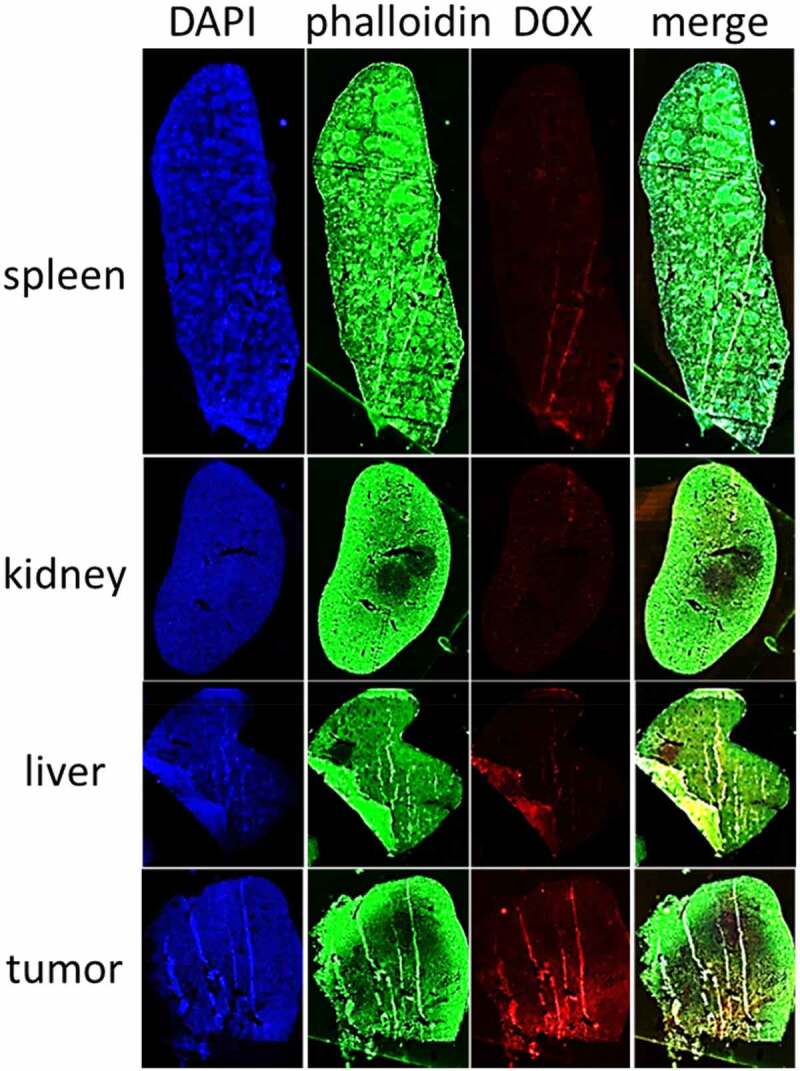
Distribution of nanoparticles with the size of 37 nm in the main tissues. All the samples were analyzed with panoramic microscope of TissueGnostics Gmbh (TissueFAXS PLUS)

In order to further visualize the distribution of nanoparticles in different tissues (liver, spleen, kidney, tumor), we selected the mice treated by nanoparticle of 37 nm at 48 h with the best tumor accumulation effect for frozen tissue section. The cellular nucleus were labeled with DAPI, and simultaneously the cytoskeleton were labeled by phalloidin. In addition, we used a panoramic microscope to shoot with a large view field, so as to determine the distribution of nanoparticles from the whole tissue and avoid misleading with local results. As shown in Figure 3, we could clearly find that there was much more nanoparticle retention in the tumor tissue than others. There was still a small amount of residual in the liver and spleen with faint red fluorescence. As the quantitative results, few nanoparticles was basically found in the kidney. All the pictures showed that the distribution of nanoparticles in tissues was not uniform, which also confirmed the necessity and rationality of panoramic microscope. The difference of nanoparticles distribution in different regions of specific tissues was being studied in our current project.

### 3.4. Small size facilitated the antitumor efficacy of nanoparticles in vivo

We have confirmed that size has a range of effects on the biological behavior of nanoparticles *in vivo*. In this study, we found that nanoparticles of appropriate small size were better for drug delivery *in vivo*. To confirm the conjecture, we established the mice model bearing U87MG tumors to verify its effectiveness in the cancer treatment. DOX was chosen as a therapeutic agent. Mice were divided into seven groups, each of five mice.

**Figure 4.**
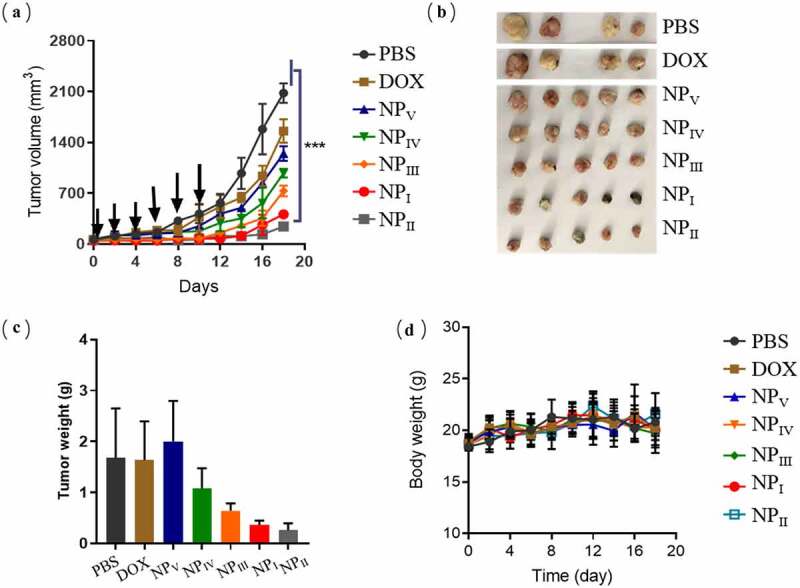
Anti-tumor activity of nanoparticles loaded with DOX. (a) Inhibition effect of DOX-loaded nanoparticles on tumor volume growth. (b) Image of tumors removed from the mice at the end of treatment. (c) The weight of tumors. (d) The changes of mice body weight during the treatment. Here was the statement that two mice were dead due to individual differences during the treatment, which were removed from the final statistics. (n = 5, data were means ± s.d.). *p < 0.05, **p < 0.01, ***p < 0.001

In the U87MG models, the equivalent of 5 mg/kg DOX-loaded nanoparticles were injected into the mice. Compared to the PBS group, the tumors in the mice treated with DOX or DOX loaded nanoparticles were all inhibited. In all the treatment groups, nanoparticles of 62 nm significantly inhibited tumor growth with an inhibition rate of 88% (Figure 4(a)). At the end of treatment, the mice were sacrificed to remove the tumors for images and weight. We could obviously observe the differences among the treatment groups in this picture, tumor volume can be effectively inhibited by the nanoparticles with the size of 62 nm (Figure 4(b)). Two mice died during the treatment due to individual differences. Considering the authenticity of the experimental data, the two mice data were not included in the statistics. The results of tumor weight measurement in mice also indicated the superiority of its therapeutic effect (Figure 4(c)). During the treatment, the body weight of the mice was monitored simultaneously, and the results showed no significant change. These could partially guarantee the safety of drug delivery system (Figure 4(d)).

### 3.5. Safety evaluation of therapeutic nanoparticles in vivo

**Figure 5.**
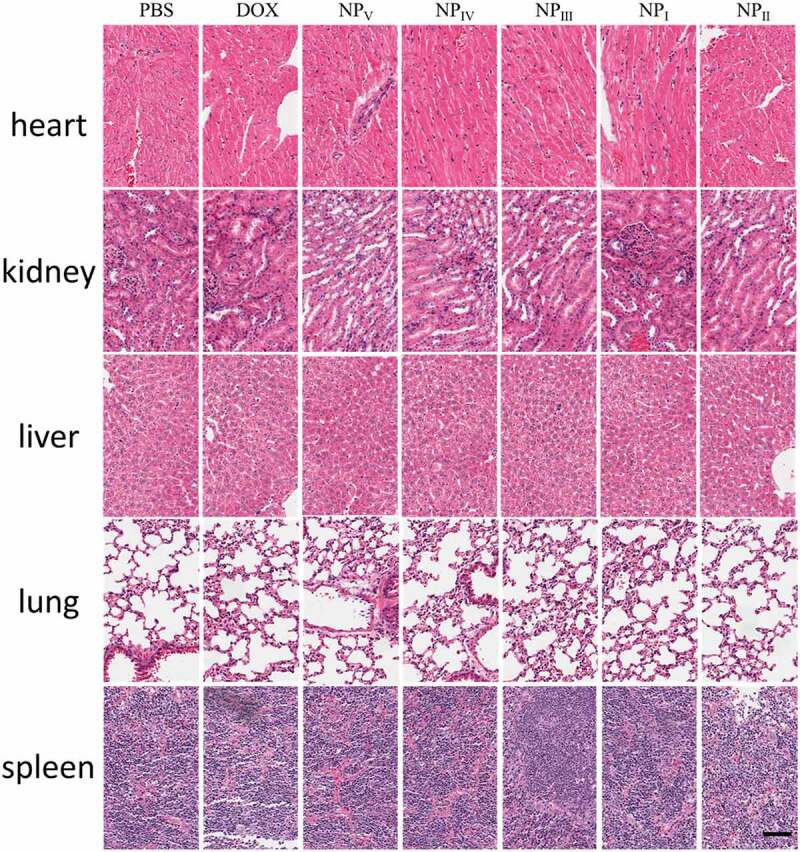
Pathological examination of main tissues. After 18 days of treatment, the tissues were collected for safe evaluation. Scale bar represent 200 μm

The therapeutic potential of nanomedicines applied *in vivo* was very important, but the safety of nanomedicines *in vivo* was also of great concern. In the treatment experiment, we indirectly demonstrated the safety of therapeutic system by monitoring the changes of mice body weight. However, the method was relatively crude, in order to investigate the effect of the therapeutic nanoparticles on the safety of mouse organs in detail. At the end of treatment, the mice were sacrificed to collect the heart, liver, spleen, lung, kidney. Part of organs were gently washed with saline, and fixed in the paraformaldehyde for 24 h. Then, they were cut into 6 μm sections and stained with hematoxylin–eosin solution. Histology of the tissues were evaluated using the microscope. As shown in Figure 5, there was no obviously inflammatory cell infiltration of mononuclear cell and neutrophil. According to the results, the state of tissues judged by the pathologist demonstrated that the therapeutic nanoparticles were safe for the mice.

## 4. Conclusion

In conclusion, we studied the effects of the size of nanoparticles on drug delivery *in vivo* through experiments of blood circulation and tissue distribution. We found that nanoparticles of small size exhibited superior circulation effects compared to larger nanoparticles. These sizes were often used in the design of nano-delivery system. In addition, we expounded the reason for the good circulation effect of small-size nanoparticles through the experiment of tissues distribution *in vivo*. Small nanoparticles seem to be better able to escape the retention of scavenging organs such as the liver. Nanoparticles of small size with long circulation effect can be more enriched in tumors. Thus, it allowed them to deliver more chemotherapeutic drugs to the tumor tissue, increasing tumor cell sensitivity to chemotherapeutic drugs. In fact, it has been well validated in cancer treatment experiment. These findings indicated that the characteristic of nanoparticles size could be as an important object for regulation, which can effectively enhance the EPR enrichment effect of nanoparticles in tumor tissues. In regard to the clinical cancer treatment, it can be used as an effective chemotherapeutic drug sensitizer and play a good role in tumor inhibition. This study provides a new therapeutic strategy for clinical cancer chemotherapy. These results will be beneficial to promote the development of nano-drugs, as well as the research of nano-vaccine, through the adjustment of size can enhance the effect of nano-drugs on tumor chemotherapy, as well as the activation effect of nano-vaccine on tumor immunity.

## Data Availability

All data produced during the current study are available from the corresponding author on reasonable request.

## Highlights

1. The regulation of nanoparticle size would trigger the sensitivity of tumor cells to chemotherapeutic drugs as a chemosensitizer.
2. The particle sizes can be controlled by adding hydrophobic polymer into the block polymer.
3. Nanoparticles of small sizes could effectively enhance the effect of chemotherapy and inhibit the growth of tumor.
